# Comparative proteomic analysis identifies biomarkers for renal aging

**DOI:** 10.18632/aging.104007

**Published:** 2020-11-06

**Authors:** Meiqi Yi, Yingying Ma, Songbiao Zhu, Chengting Luo, Yuling Chen, Qingtao Wang, Haiteng Deng

**Affiliations:** 1 MOE Key Laboratory of Bioinformatics, Center for Synthetic and Systematic Biology, School of Life Sciences, Tsinghua University, Beijing, China; 2 Beijing Chaoyang Hospital Affiliated to Capital Medical University, Beijing, China

**Keywords:** renal aging, proteomics, biomarkers, glutathionylation, NMN

## Abstract

Proteomics have long been applied into characterization of molecular signatures in aging. Due to different methods and instrumentations employed for proteomic analysis, inter-dataset validation needs to be performed to identify potential biomarkers for aging. In this study, we used comparative proteomics analysis to profile age-associated changes in proteome and glutathionylome in mouse kidneys. We identified 108 proteins that were differentially expressed in young and aged mouse kidneys in three different datasets; from these, 27 proteins were identified as potential renal aging biomarkers, including phosphoenolpyruvate carboxykinase (Pck1), CD5 antigen-like protein (Cd5l), aldehyde dehydrogenase 1 (Aldh1a1), and uromodulin. Our results also showed that peroxisomal proteins were significantly downregulated in aged mice, whereas IgGs were upregulated, suggesting that peroxisome deterioration might be a hallmark for renal aging. Glutathionylome analysis demonstrated that downregulation of catalase and glutaredoxin-1 (Glrx1) significantly increased protein glutathionylation in aged mice. In addition, nicotinamide mononucleotide (NMN) administration significantly increased the number of peroxisomes in aged mouse kidneys, indicating that NMN enhanced peroxisome biogenesis, and suggesting that it might be beneficial to reduce kidney injuries. Together, our data identify novel potential biomarkers for renal aging, and provide a valuable resource for understanding the age-associated changes in kidneys.

## INTRODUCTION

The world’s elderly population continues to grow at an unprecedented rate, especially in developed countries. Estimates predict an increase in life expectancy to 88 years for men and to 91 years for women in 2030 [1]. With the rapidly growing elderly population comes the need to better understand the mechanisms of aging. However, since not all organs age at the same pace, it is important to characterize the aging process for each organ. Renal aging involves molecular, cellular, structural, and functional changes, leading to susceptibility to environmental and internal stresses. Age-associated modifications of kidneys include changes in glomerular structure, such as glomerular basement membrane thickening or wrinkling associated with loss of capillary loops, and mesangial matrix expansion [2, 3]. The reduced renal function in aging is often accompanied by a progressive decline in glomerular filtration rate, tubular dysfunction, decreased sodium reabsorption, potassium excretion, and urine concentrating capacity [4]. Consequently, these changes compromise the ability of kidneys to withstand environmental insults and injury, leading to the high susceptibility of aged population to acute kidney injury [5, 6] and chronic kidney disease [7, 8]. A comprehensive profile of the molecular changes during aging is the key to understand the mechanisms of the aging process and to develop therapeutic interventions.

Over the last two decades, mass spectrometry (MS)-based proteomics has been widely used to identify differentially expressed proteins between young and aged populations [9, 10]. Earlier studies that employed two-dimensional electrophoresis (2-DE) usually identified several hundreds of proteins that only covered a small portion of the proteome, and their quantification was largely based on label-free methods [11]. Recently, the gel-free based analysis and stable isotope coded labeling have become the mainstream approaches allowing for accurate identification and quantitation of thousands of proteins. Using protein quantitation by stable isotope labeling with amino acids in cell culture (SILAC) [12], Mann et al. found that expression levels of the vast majority of proteins remained virtually unchanged during aging in mouse kidneys [13], suggesting that the proteome is efficiently maintained even at a relatively high age. A recent study using multi-omics analysis showed that aldehyde dehydrogenase 1 (Aldh1a1) was one of the most upregulated proteins in aged mouse kidneys [14]. In addition, ceramidase (Asah1) was highly expressed in aged kidneys, consistent with a decrease in ceramide C16 [14]. These studies provided valuable information about the protein changes during renal aging, but the variations between different datasets need to be inter-validated. It has long been recognized that accumulation of protein oxidative damage contributes to aging and diseases. Contrary to the popular belief that the levels of reactive oxygen species (ROS) increase during aging, Haopeng et al. recently showed that global cysteine modification did not increase with age, and found a complex and tissue specific remodeling of cysteine oxidation during aging [15].

In this study, we performed proteomics analysis of young and aged mouse kidneys, and compared our results with two other proteomics datasets. Using this approach, we identified a set of proteins that were differentially expressed in young and aged kidneys, and might thus serve as biomarkers for renal aging. Our data indicate that peroxisomes are the major targets of renal aging, and that nicotinamide mononucleotide (NMN) administration effectively enhances peroxisome biogenesis. In addition, our results show that peroxisomal proteins and fatty acid oxidation-related proteins are glutathionylated in aged mice.

## RESULTS

### Identification of differentially expressed proteins in young and aged mouse kidneys by comparative analysis of proteomics datasets

**Figure 1:**
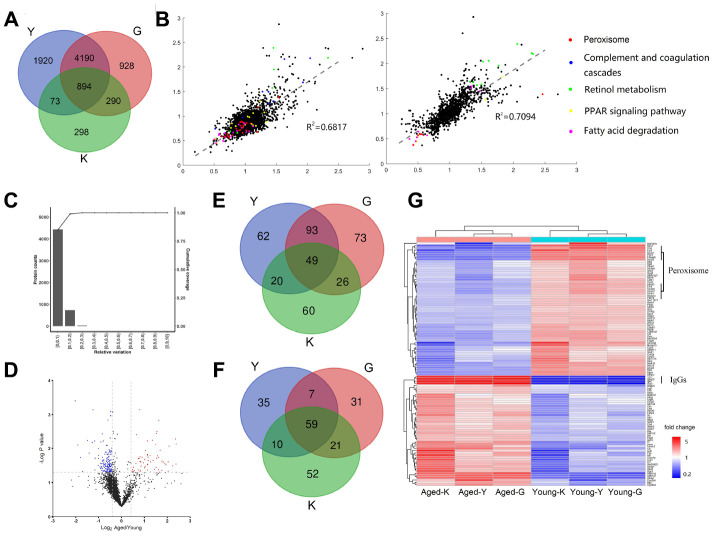
**Differentially expressed proteins (DEPs) in young and aged mouse kidneys.** (**A**) Comparison of three proteomic datasets: this study and (14, 16). (**B**) Scatter plots showing correlations between our data to G-dataset (left) and to K-dataset (right) (Red dot, Peroxisome; Blue dot, Complement and coagulation cascades; Green dot, Retinol metabolism; Yellow dot, PPAR signaling pathway; Pink dot, Fatty acid degradation). (**C**) Determination of the cut-off thresholds for DEPs. (**D**) Volcano diagram of DEPs between aged and young mouse kidneys. (**E**, **F**) Venn diagram comparing the up-regulated (**E**) and down-regulated (**F**) proteins in this study and (14, 16). (**G**) Hierarchical clustering of DEPs presented in all three datasets.

To investigate the proteome changes in young (8 weeks, n=6) and aged (96 weeks, n=6) mouse kidneys, we carried out a comprehensive proteomic analysis using Tandem Mass Tag (TMT)-based quantitation. In total, 7,208 proteins were identified from three biological replicates with a tight correlation, indicating that these data were highly reproducible. Our data (labeled as Y) were then compared to those by Gygi et al. (labeled as G-dataset) [16] and Kurschat et al. (labeled as K-dataset) [14], as shown in Venn Diagram in Figure 1A. 6302 proteins were identified in G-dataset [16], from which 5084 proteins (80%) were also present in our dataset (Figure 1A), indicating that about 80% proteins were present in both datasets. Kurschat dataset identified only 1555 proteins, from which 967 proteins (62%) were present also in our dataset. These results demonstrated that more than 60% of proteins were consistently identified in three different laboratories. Scatter plot of any two datasets exhibited a tight correlation, indicating that these data were highly similar (R^2^ = 0.7; Figure 1B).

The statistical significance threshold cutoff for identifying differentially expressed proteins (DEPs) was determined by population statistics (Figure 1C) [17, 18]. Volcano diagram showed that 224 proteins were upregulated (Aged/Young ratio > 1.5 and FDR adjusted p < 0.05; Supplementary Table 1) and 111 proteins were downregulated (Aged /Young ratio < 0.67 and FDR adjusted p < 0.05; Supplementary Table 2) (Figure 1D). About 64% of the upregulated proteins and 59% of the downregulated proteins were also identified in G-dataset (Figure 1E and 1F). Overall, 49 upregulated proteins and 59 downregulated proteins were present in all three datasets; these proteins were considered as the differentially expressed proteins between young and aged mice. The heat-map showed that among the 108 identified DEPs, IgGs and complement components exhibited a significant increase in aged mouse kidneys (Figure 1G and Supplementary Table 1).

**Table 1:** The biomarkers for renal aging.

**Accession**	**Gene Name**	**Description**	**Ratio (Aged/Young)**	**Plasma Protein**	**Urine Protein**	**High Expression in Kidney**
Q9Z2V4	Pck1	Phosphoenolpyruvate carboxykinase, cytosolic [GTP]	0.58	Y	Y	Y
P12658	Calb1	Calbindin	0.71	Y	Y	Y
P32020	Scp2	Non-specific lipid-transfer protein	0.72	Y	N	Y
P97494	Gclc	Glutamate-cysteine ligase catalytic subunit	0.72	Y	Y	Y
P24270	Cat	Catalase	0.70	Y	Y	Y
P06281	Ren1	Renin-1	0.58	Y	Y	Y
Q64433	Hspe1	10 kDa heat shock protein	0.73	Y	Y	Y
Q9JKR6	Hyou1	Hypoxia up-regulated protein 1	0.76	Y	Y	N
A2A7A7	H6pd	GDH/6PGL endoplasmic bifunctional protein	1.36	Y	N	N
Q91X72	Hpx	Hemopexin	1.44	Y	Y	N
P13597	Icam1	Intercellular adhesion molecule1	1.26	Y	Y	Y
P09813	Apoa2	Apolipoprotein A-II	1.75	Y	Y	N
P30115	Gsta3	Glutathione S-transferase A3	1.51	Y	Y	N
Q00623	Apoa1	Apolipoprotein A-I	1.34	Y	Y	N
P46412	Gpx3	Glutathione peroxidase 3	1.91	Y	Y	Y
P18242	Ctsd	Cathepsin D	1.49	Y	Y	Y
Q07797	Lgals3bp	Galectin-3-binding protein	1.66	Y	Y	N
Q549A5	Clu	Clusterin	2.00	Y	Y	Y
P21614	Gc	Vitamin D-binding protein	2.25	Y	Y	N
P08226	Apoe	Apolipoprotein E	1.93	Y	Y	Y
A2ATU0	Dhtkd1	Probable 2-oxoglutarate dehydrogenase E1 component DHKTD1	1.90	Y	N	Y
O70570	Pigr	Polymeric immunoglobulin receptor	2.23	Y	Y	Y
P29788	Vtn	Vitronectin	2.02	Y	Y	N
Q91X17	Umod	Uromodulin	2.14	Y	Y	Y
P24549	Aldh1a1	Retinal dehydrogenase 1	2.56	Y	Y	Y
Q8K1I3	Spp2	Secreted phosphoprotein 24	2.52	Y	Y	N
Q9QWK4	Cd5l	CD5 antigen-like	3.33	Y	Y	N

### Determination of biomarkers for renal aging by proteomic profiling

**Figure 2:**
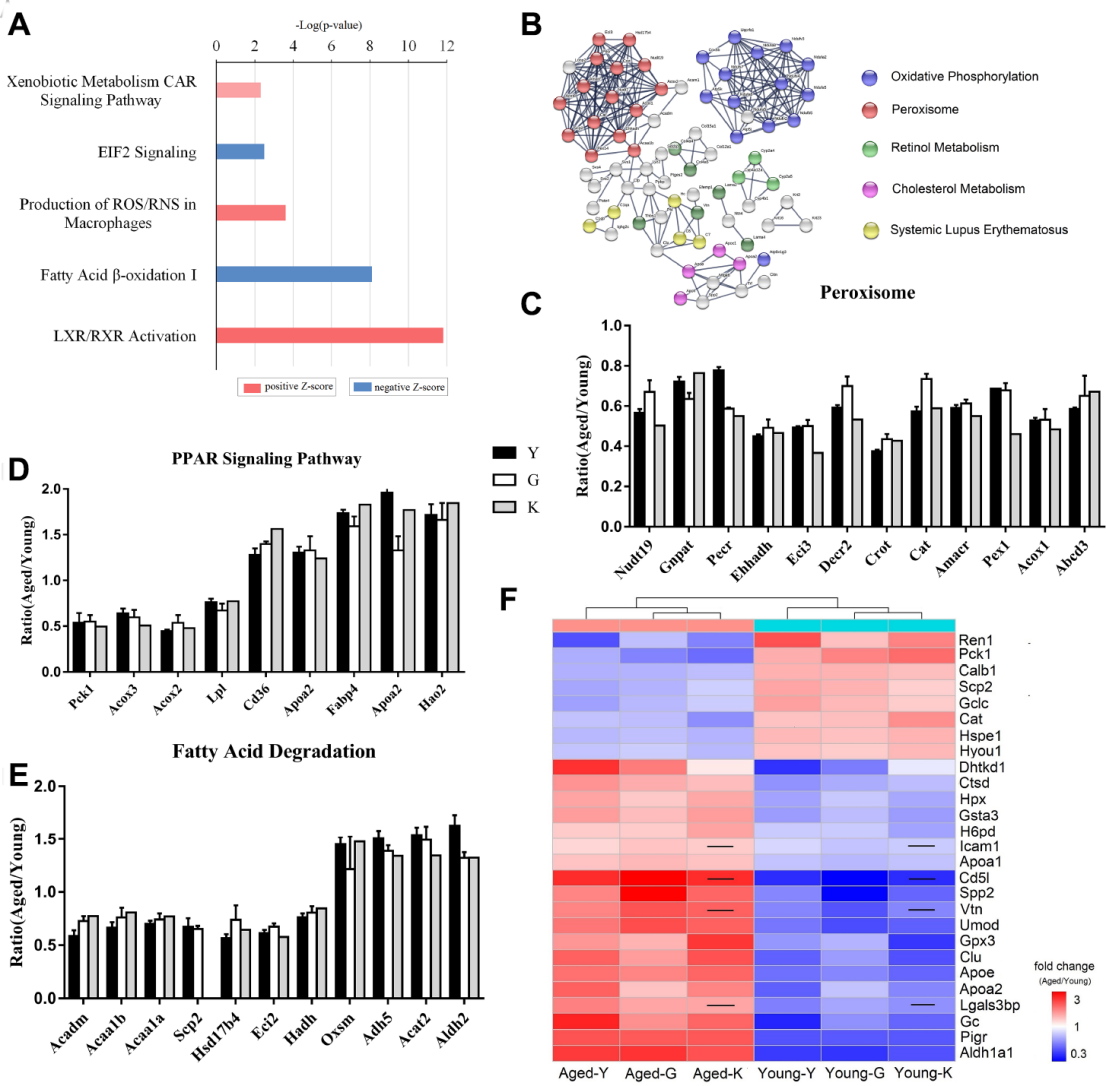
**Biomarkers for renal aging.** (**A**) Ingenuity Pathway Analysis (IPA) of DEPs in aged and young mouse kidneys analyzed by proteomics. (**B**) STRING network analysis based on robust Spearman correlation for DEPs in aged and young mouse kidneys. (**C**–**E**) Histogram of peroxisomal proteins (**C**), proteins associated with PPAR signaling (**D**), and proteins associated with fatty acid degradation pathway (**E**). (**F**) A heat-map showing biomarkers for renal aging; the black line indicates that the corresponding protein was not present in K-dataset. Error bars represent ± SEM.

Ingenuity Pathway Analysis (IPA) of the 108 DEPs showed that these proteins were highly enriched in oxidative phosphorylation pathway, sirtuin signaling, fatty acid β-oxidation pathway, and bile acid biosynthesis pathway. Specifically, the liver X receptors/retinoid X receptor (LXR/RXR) pathway, production of nitric oxide and ROS in macrophages, and the xenobiotic metabolism CAR signaling pathway were activated, while EIF2 signaling and fatty acid β-oxidation were inactivated (Figure 2A). String analysis showed that multiple proteins associated with oxidative phosphorylation, peroxisomes, retinol metabolism, and cholesterol metabolism were differentially expressed in young and aged mice (Figure 2B). All three datasets showed that peroxisome-associated proteins were downregulated in aged mice (Figure 2C). Peroxisomes contain enzymes that catalyze metabolic reactions, such as oxidation of very long-chain fatty acids, synthesis of ether lipids and bile-acid, and degradation of reactive oxygen and nitrogen species [19]. According to the proteomic results, all enzymes associated with fatty acid oxidation were downregulated; the peroxisomal carnitine O-octanoyltransferase (Crot) was decreased by more than 50%. Unlike peroxisomal proteins, which were uniformly decreased in aged mice, proteins associated with PPAR signaling were either up- or down-regulated in aged mice (Figure 2D). Besides, phosphoenolpyruvate carboxykinase 1 (Pck1) was downregulated by 50%, while CD36 was upregulated. Among proteins related to fatty acid degradation, 3-oxoacyl-ACP synthase (OXSM) was also upregulated (Figure 2E). The other identified differentially expressed proteins, Ahsg, Alb, Apoe, C3, Cat, Clu, Gc, Hpx, Ren, and Slc22a6 are associated with renal damage and renal tubule injury [20–29].

To identify potential biomarkers for renal aging, we excluded proteins associated with subcellular organelles and abundant plasma proteins. Specifically, we considered proteins that are present in plasma and/or urine, and are highly expressed in renal tissues as biomarkers. Using these criteria, we selected 27 proteins as potential biomarkers for renal aging (Figure 2F and Table 1); expression of 19 proteins was increased, while expression of 8 proteins was decreased in aged kidneys. These proteins participated in 5-hydroxytryptamine degradation, angiogenesis, blood coagulation, pyruvate metabolism, TCA cycle, and vitamin D metabolism (Supplementary Table 3).

### Redox proteomics shows high protein glutathionylation in aged mouse kidneys

**Figure 3:**
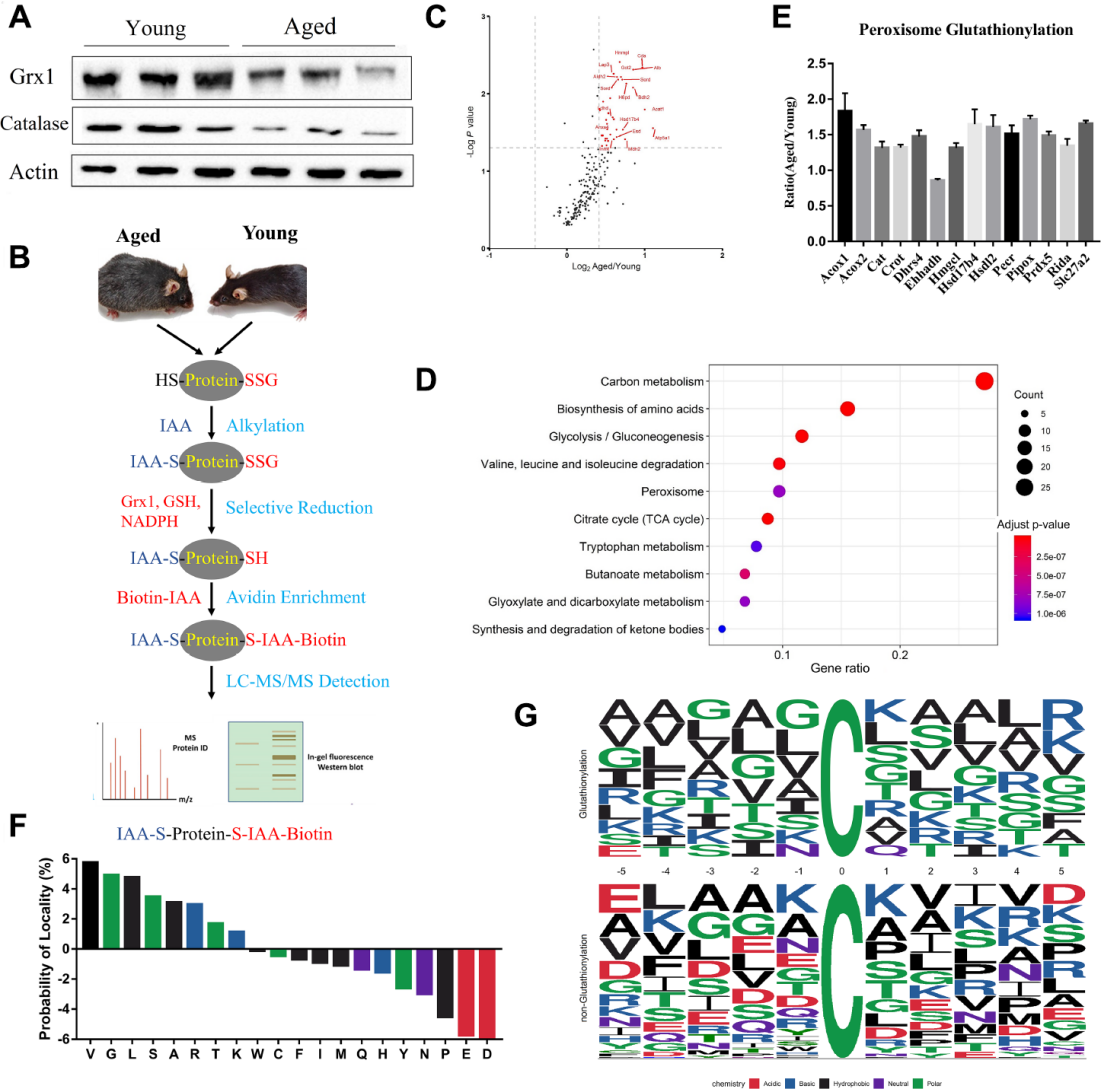
**Profiling protein glutathionylation in young and aged mouse kidneys.** (**A**) Western blot analysis of Glrx1 and catalase in kidney tissues. (**B**) Workflow of profiling cysteine glutathionylation. (**C**) Volcano plot of glutathionylated proteins in young and aged kidneys; glutathionylated peroxisomal proteins are red-coded. (**D**) KEGG enrichment analysis of glutathionylated proteins. (**E**) Histogram of the increased glutathionylation of peroxisomal proteins. (**F**) Significant enrichment of small amino acids, lysine and arginine against aspartic acid and glutamic acid at the proximal position (±five positions) to glutathionylated cysteine residues. (**G**) Consensus motif for glutathionylated cysteine residues shows significant enrichment of lysine and arginine across a range of proximal positions. Error bars represent ± SEM.

It has been amply documented that oxidative stress increases in aged population [30, 31]. Our proteomics and western blotting data showed that catalase expression was downregulated in aged kidneys, indicating increased ROS levels (Figure 3A). Furthermore, we found that glutaredoxin-1 (Glrx1) expression was downregulated, using both proteomics and western blotting analyses. Glrx1 is an oxidation repair enzyme that regulates protein mixed disulfides through deglutathionylating an S-glutathionylated substrate [32]. The results proposed that protein glutathionylation was increased in aged mouse kidneys. To confirm it, we performed an analysis of glutathionylated proteins. The experimental procedure to enrich glutathionylated peptides was based on selective deglutathionylation by Glrx1. Briefly, proteins extracted from kidney tissues were treated with iodoacetamide to modify the free sulfhydryl groups, followed by deglutathionylation by Glrx1. The newly generated free sulfhydryl groups were then alkylated with biotin-IAA, enriched by streptavidin affinity column, and analyzed by LC-MS/MS to determine potential glutathionylation sites (Figure 3B). Using this approach, 342 unique glutathionylated peptides from 281 proteins were identified. Interestingly, almost all identified proteins exhibited increased glutathionylation (Figure 3C).

KEGG analysis revealed that proteins with increased glutathionylation were associated with carbon metabolism, amino acid metabolism, and peroxisome pathways in aged mice (Figure 3D). Quantitative proteomics showed that peroxisomal proteins were downregulated, and glutathionylome analysis revealed that peroxisomal proteins were highly glutathionylated (Figure 3E). Further analysis revealed that lysine and arginine residues were highly enriched against aspartic acid and glutamic acid at the proximal positions (±5) to glutathionylated cysteine residues. Consensus sequence for glutathionylation motif showed enrichment of arginine against aspartic acid and glutamic acid across a range of proximal positions (Figure 3F and 3G). We also found that glycine (G), alanine (A), valine (V), and leucine (I), which are small and nonpolar amino acids, frequently appeared in the proximity of glutathionylated residues.

### NMN rescues proteostasis in aged mouse kidneys

**Figure 4:**
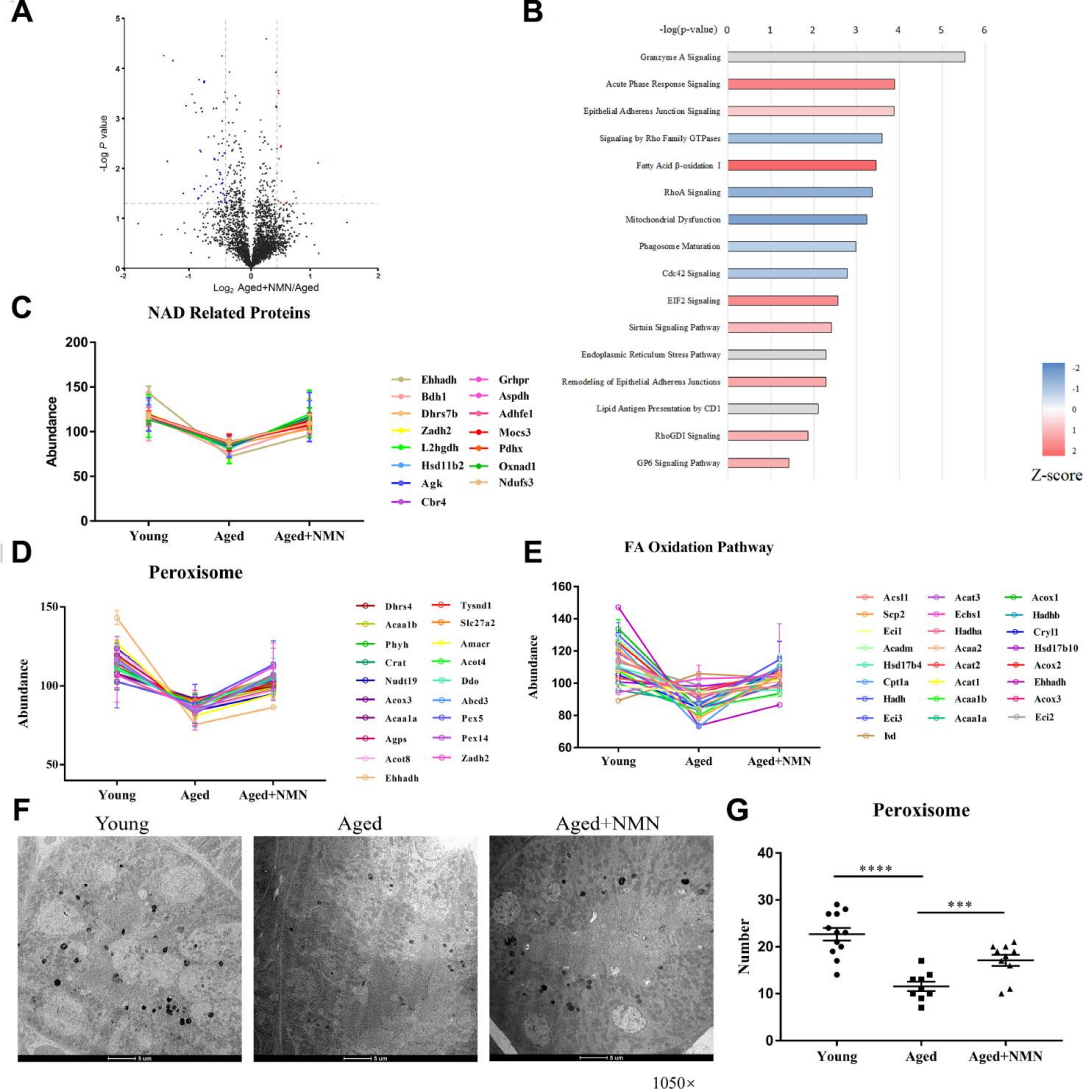
**NMN restores proteostasis in aged kidneys.** (**A**) Volcano plot of DEPs in untreated and NMN-treated kidneys. (**B**) IPA analysis of DEPs in untreated and NMN-treated kidneys (p < 0.05). (**C**) Expression of NAD-dependent oxidoreductase. (**D**–**E**) Expression of proteins associated with peroxisomes (**D**) and fatty acid oxidation pathways (**E**). (**F**) Electron micrograph showing part of the mouse kidneys stained for catalase by DAB. The electron-dense reaction product of DAB is observed in the matrix of all peroxisomes, while mitochondria and other components of the cytoplasm are not stained. (Mag. ×1,050). (**G**) The number of peroxisomes in young, aged and NMN-treated kidneys based on electron micrographs. Data were analyzed using Student’s t-test. *p < 0.05, **p < 0.01, and ***p < 0.001. p < 0.05 was considered statistically significant. Error bars represent ± SEM.

Recent studies have shown that nicotinamide mononucleotide (NMN) supplementation may ameliorate age-associated organ dysfunction [33, 34]. To examine whether NMN treatment could rescue aged-impaired proteostasis, we treated aged mice (96 weeks) with NMN (500 mg/kg body weight, every two days for 4 weeks), and analyzed the kidney tissues of untreated and NMN-treated mice by quantitative proteomics. NMN treatment upregulated 190 proteins, while it downregulated 134 proteins in kidneys (Figure 4A). In addition, our results showed that NMN reversed proteostasis compared to untreated aged mice. IPA analysis showed that proteins associated with oxidative phosphorylation, sirtuin signaling, fatty acid β-oxidation, and bile acid biosynthesis pathway were upregulated in NMN-treated mice (Figure 4B). Moreover, we found that peroxisomal proteins, NAD(H)-related proteins, and proteins associated with fatty acid β-oxidation pathway were increased after NMN treatment (Figures 4C–4E).

To determine whether NMN increases peroxisome biogenesis, we analyzed mouse kidneys by electron microscopy, using specific peroxisome labeling by diaminobenzidine (DAB) (Figure 4F). Our results showed that the number of peroxisomes significantly decreased in aged mouse kidneys, but NMN efficiently increased the number of peroxisomes (Figure 4G). NMN supplementation also decreased IgGs and complement protein levels, suggesting that NMN might reduce kidney injuries (data not shown).

## DISCUSSION

Although proteomic analysis has been considered an ideal approach to decipher the proteome landscape in aging, we have not generated the consistent results allowing us to define biomarkers for aging. In this study, we performed proteomic analysis of kidney tissues from young and aged mice, and compared our data to datasets provided by earlier studies (G- and K-datasets). A previous report (K-dataset) identified 1555 proteins from mouse kidneys; 967 of these proteins (70%) were also present in our data (Figure 1A), indicating that our proteomic analysis for identification of 7208 proteins reached a high degree of proteome coverage. Among the identified 7208 proteins in our study, 224 proteins were upregulated; from these, 102 proteins were also present in G-dataset and 69 proteins were present in K-dataset. We also identified 111 downregulated proteins; 66 proteins were present in G-dataset, and 69 proteins were present in K-dataset. Overall, there were 108 proteins differentially expressed in the three datasets. After excluding proteins localized in subcellular compartments, we identified 27 proteins as potential biomarkers for renal aging; these proteins were also present in the human plasma database.

Among the proteins upregulated in aged mice, Aldh1a1 was found highly expressed in the medullary thick ascending limb segment of the loop of Henle [14]. Aldh1a1 is associated with drug metabolism and retinoic acid signaling by converting retinaldehyde to retinoic acid [35, 36]. Although mechanisms underlying the high expression of Aldh1a1 in aged mice remain elusive, it is thought that the retinoic acid levels are high in aged kidney, resulting in activation of the RXR/PPAR pathway. Thus, we suggest that Aldh1a1 may serve as a potential target for aging intervention, and that the FDA-approved drug disulfiram should be tested in future clinical trials [37].

On the other hand, Pck1 was identified as the most down-regulated protein in aged kidneys. Pck1 catalyzes conversion of oxalacetate to phosphoenolpyruvate, and participates in gluconeogenesis, glyceroneogenesis, and synthesis of serine [38]. Pck1 was a known aging marker, whose overexpression in skeletal muscle rendered mice seven times more active in their cages than controls. These mice ate more, but maintained low body weight. Importantly, these mice had an extended life span relative to control animals [39]. Therefore, we propose that Pck1 activation may increase renal functions in aged mice.

Two anti-oxidant enzymes catalase (Cat) and glutathione peroxidase-3 (Gpx3) were present in our biomarker list. Cat and Gpx3 constitute a part of cellular defense mechanism against oxidation [40, 41]. Intriguingly, Cat was downregulated, while Gpx3 was upregulated in aged mouse kidneys. Altered renal levels of *CAT* and *GPX* mRNA were found in kidneys before development of hypertension in spontaneously hypertensive rats. Thus, future studies should elucidate the precise roles of these anti-oxidant enzymes in aging.

Protein glutathionylation is important in anti-oxidative stress response; it modulates protein function and degradation, which also participates in aging process [42, 43]. Interestingly, previous studies (G-dataset) indicated that global cysteine modification did not increase with age [16]. We found that protein glutathionylation was significantly increased in aged mice; this was consistent with Glrx1 downregulation in aged mice (Figure 3A). We also noticed that age increased glutathionylation of peroxisome-related proteins. Peroxisomes are cell organelles that can cause disease if they do not function properly. Extensive studies indicate the involvement of peroxisomes in the pathogenesis of kidney injury [44, 45]. However, further studies are needed to elucidate the molecular mechanisms induced by dysfunctional peroxisomes in renal aging. Our TEM data showed that the number of peroxisomes was significantly reduced during aging, and this reduction could be reversed by NMN treatment.

In addition, we found that 16 out of 19 highly expressed proteins in aged mice were downregulated in NMN-treated aged mice, while 6 out of 8 under-expressed proteins were upregulated in NMN-treated aged mice. Briefly, expressions of 22 age markers, including Pck1, Aldh1a1, Gpx3, Cat and Umod were reciprocally regulated by NMN treatment in aged mice. It has been amply documented that NMN treatment increased cellular NAD^+^ level and promoted mitochondrial biogenesis. Consistently, NMN treatment upregulates 10 kDa Heat Shock Protein that is a mitochondrial chaperonin. Aldh1a1 catalyzes NAD^+^-dependent dehydrogenation and increasing NAD^+^ may downregulate Aldh1a1 and H6pd expressions. It is known that NAD^+^ regulates cell redox homeostasis, and consistently, we reveal that NMN treatment upregulates proteins associated with glutathione metabolism pathway such as Gclc, but it downregulates Gpx3 and Gst3a. Noticeably, NMN treatment had no effects on expression of lipid transporting-associated proteins such as Scp2, Apoa1 and Apoe (Supplementary Table 4).

In summary, using comparative proteomics analysis, we identified a set of proteins that were differentially expressed in young and aged mouse kidneys, and might thus serve as biomarkers for renal aging. One of the most upregulated proteins in aged kidneys was Aldh1a1, suggesting that it might serve as a potential target for aging interventions. In addition, our results showed that aging caused a reduction in peroxisomes, and that NMN administration restored the peroxisome homeostasis, suggesting that NMN supplementation might be beneficial for kidney health. Our data provide a valuable resource for understanding the age-associated changes in kidneys, and reaffirm that proteomics is a powerful approach in aging studies.

## MATERIALS AND METHODS

### Animals and animal care

C57BL/6J mice were housed at the Laboratory Animal Research Center, Tsinghua University. Mice were intraperitoneally injected with nicotinamide mononucleotide (NMN) at 500 mg/kg body weight every two days for 4 weeks. Untreated and NMN-treated mice, 8- and 96-weeks-old, were sacrificed for further analyses. Mouse renal tissues were collected and washed in cold PBS, and immediately snapped frozen in liquid nitrogen for the following study including the biochemical, proteomics and metabolomics analyses. The remaining tissues were fixed in 4% paraformaldehyde for further histopathological examination.

### Quantitative proteomics analysis

Proteins were extracted from kidney tissues by 8 M urea containing 1% protease inhibitor mix. Protein (200 μg) disulfides were reduced by 5 mM Tris (2-carboxyethyl) phosphine (TCEP) for 10 min at room temperature. Then, free cysteine residues were alkylated by 10 mM iodoacetamide for 30 min in the dark. After dilution with PBS, proteins were digested with sequencing-grade trypsin for 16 h at 37 °C at a substrate/enzyme ratio of 50:1 (w:w). Peptides were desalted using Sep-Pak C18 Cartridge (Waters, 186004619), vacuum dried, and labeled with Tandem Mass Tag (TMT, Thermo, Waltham, MA). Mixed peptides were fractionated using the high-pH reversed phase chromatography followed by nano LC-MS/MS analysis.

The MS/MS spectra were searched against the UniProt mus musculus database (June 24, 2017; 59,594 sequences) processed by the Sequest HT search engine using Proteome Discoverer (PD) 2.1 software. The following parameters were used for searching: fixed modifications of carbamidomethylation on cysteine and TMT 6-plex on lysine and peptide N-terminus; variable modification of oxidation on methionine; two trypsin missed cleavages were permitted; the tolerances of precursor and fragment mass were 10 ppm and 0.02 Da; at least two unique peptides for identification of proteins; and 1% FDR (false discovery rate) tolerance at peptide-spectrum match (PSM) level.

### Glutathionylation enrichment assay

Tissue samples were homogenized and lysed by 8 M urea containing 1% protease inhibitor mix; 10 mg protein samples were used for enrichment of glutathionylated peptides. Briefly, the lysates were alkylated by iodoacetamide without reduction, and digested with sequencing-grade trypsin for 16 h at 37 °C. After digestion, samples were incubated with Glrx1 in the presence of GSH, NADPH, and GR (glutaredoxin reductase, Sigma). Biotin-IAA (Thermo Fisher) was used to alkylate the newly generated sulfhydryl groups. The products were incubated with streptavidin beads (Thermo Fisher), eluted by 1% trifluoroacetic acid solution, and labeled by Tandem Mass Tag (TMT).

### Western blotting analysis

Tissue samples were lysed in RIPA buffer (Solarbio, Beijing, China) containing 1% Protease Inhibitor Cocktail (Selleck, Shanghai, China). The lysates were centrifuged at 12,000 rpm for 20 min; the protein concentration was determined by the BCA protein assay (Solarbio, Beijing, China). Equal amounts of protein were separated by SDS-PAGE, and transferred to PVDF membranes. After 1 h blocking in TBST containing 5% skim milk, primary and secondary antibodies were used to detect the target proteins. Immunoblots were processed through the enhanced chemiluminescence (ECL); the images were quantified using ImageLab software. Actin was used as an internal loading control. Primary and secondary antibodies were the following: anti-Glrx1 antibody was purchased from Abcam (1:1000 diluted, ab187507); anti-actin antibody was from Cell Signaling Technology (1:50000 diluted, 4970); anti-rabbit-IgG antibody was purchased from Cell Signaling Technology (1:2000 diluted, 7074S).

### Cytochemical detection of peroxisomes by transmission electron microscopy

Mice were sacrificed and one of the kidneys was removed and fixed by glutaraldehyde-formaldehyde overnight at 4 °C. After fixation, the tissue was rinsed in PBS and sectioned into 200 μm slices by the Oxford Vibratome (Foster City, CA, USA). The 200 μm sections were pre-incubated (1 h, room temperature) in DAB medium (Proteintech, Chicago, US) without hydrogen peroxide, followed by incubation (1 h) in a complete medium containing 0.15% hydrogen peroxide. After incubation, the sections were rinsed with PBS, and re-fixed in glutaraldehyde-formaldehyde fixative. After dehydration in chilled graded ethanol solutions and embedding in Epon by propylene oxide for 12-16 h, ultrathin sections were cut by an ultra-microtome, and examined by electron microscope.

### Statistics

GraphPad Prism (version 6.0) and R (version 3.5.2) programs were used for statistical analyses. All experiments were repeated three times and statistical differences were assessed by two-tailed unpaired Student’s *t* test and considered significant at *p* ≤ 0.05 (*, p < 0.05; **, p < 0.01; ***, p < 0.001). Data were expressed as the mean ± SEM (standard error of mean). For further evaluation, differentially expressed proteins in proteomics study were analyzed by Ingenuity Pathway Analysis (IPA, QIAGEN) to reveal their potential relationships and intracellular pathways. Gene Ontology (GO) and Kyoto Encyclopedia of Genes and Genomes (KEGG) analyses for the identified proteins were performed to construct a network with related proteins.

## Supplementary Material

Supplementary Table 1

Supplementary Table 2

Supplementary Tables 3 and 4
