# Supplementary material for: Comparative proteomic analysis identifies biomarkers for renal aging
Source: Aging (Albany NY). 2020 Nov 6;12(21):21890–903. doi: 10.18632/aging.104007 (PMC7695359; doi:10.18632/aging.104007)
Supplement: Supplementary Table 1 [file aging-12-104007-s001..docx]

**Supplementary Table 1. The upregulated proteins in aged kidney.**

| Accession | Gene Symbol | Kidney Young | Kidney  Aged | p-value | Description |
| --- | --- | --- | --- | --- | --- |
| Q3UEQ9 | Aass | 78.30 | 121.70 | 2.24E-03 | Aminoadipate-semialdehyde synthase |
| E9QMV2 | Abracl | 77.97 | 122.00 | 5.67E-02 | Costars family protein ABRACL |
| Q05117 | Acp5 | 79.20 | 120.80 | 2.34E-02 | Tartrate-resistant acid phosphatase type 5 |
| A0A3B0ITG8 | Adia | 52.73 | 147.27 | 5.09E-02 | Adiponectin a |
| A0A3B0INZ4 | Adib | 56.30 | 143.70 | 2.98E-02 | Adiponectin b |
| O89020 | Afm | 80.00 | 120.00 | 3.77E-04 | Afamin |
| Q60F90 | Ahsp | 75.43 | 124.57 | 1.98E-02 | Alpha-hemoglobin stabilizing protein |
| Q571M4 | Akr1c13 | 76.03 | 123.93 | 4.75E-04 | MKIAA4014 protein (Fragment) |
| Q546G4 | Alb | 79.27 | 120.77 | 6.02E-03 | Albumin 1 |
| P24549 | Aldh1a1 | 55.23 | 144.77 | 2.03E-07 | Retinal dehydrogenase 1 |
| O35945 | Aldh1a7 | 57.20 | 142.83 | 4.26E-04 | Aldehyde dehydrogenase, cytosolic 1 |
| Q8R0Z6 | Angptl6 | 45.37 | 154.60 | 9.01E-03 | Angiopoietin-related protein 6 |
| B7STB7 | Anxa1 | 79.47 | 120.50 | 9.27E-03 | Annexin |
| A7YL62 | Apoa2 | 75.30 | 124.73 | 1.72E-02 | Apolipoprotein A-II |
| Q8VBT6 | Apobr | 77.60 | 122.40 | 5.71E-04 | Apolipoprotein B receptor |
| P34928 | Apoc1 | 77.37 | 122.63 | 1.01E-02 | Apolipoprotein C-I |
| Q61268 | Apoc4 | 79.10 | 120.90 | 4.51E-02 | Apolipoprotein C-IV |
| P08226 | Apoe | 62.07 | 137.90 | 6.67E-04 | Apolipoprotein E |
| Q01339 | Apoh | 74.63 | 125.37 | 8.21E-03 | Beta-2-glycoprotein 1 |
| Q3TZR9 | Atf7 | 65.10 | 134.90 | 3.07E-02 | Activating transcription factor 7 |
| A0A338P7G3 | Atp5o | 72.77 | 127.23 | 1.18E-02 | ATP synthase, H+ transporting, mitochondrial F1 complex |
| Q64726 | Azgp1 | 77.47 | 122.53 | 1.26E-03 | Zinc-alpha-2-glycoprotein |
| P28653 | Bgn | 67.00 | 133.00 | 3.29E-03 | Biglycan |
| Q3TAF9 | Bgn | 70.20 | 129.80 | 1.68E-02 | Biglycan |
| S4R270 | Bin2 | 74.03 | 126.00 | 6.40E-04 | Bridging integrator 2 |
| P07743 | Bpifa2 | 46.13 | 153.87 | 1.39E-04 | BPI fold-containing family A member 2 |
| P98086 | C1qa | 55.83 | 144.17 | 2.16E-02 | Complement C1q subcomponent subunit A |
| D3YZ61 | C1qtnf3 | 72.63 | 127.37 | 2.51E-02 | Complement C1q tumor necrosis factor-related protein 3 |
| Q566I6 | C1ra | 74.47 | 125.50 | 1.82E-02 | Complement component 1, r subcomponent |
| B2RWX2 | C4b | 73.27 | 126.73 | 3.76E-04 | Complement component 4B (Childo blood group) |
| P08607 | C4bpa | 66.40 | 133.60 | 3.59E-04 | C4b-binding protein |
| P06684 | C5 | 60.37 | 139.67 | 5.79E-04 | Complement C5 |
| Q91X70 | C6 | 66.00 | 134.00 | 3.72E-05 | Complement component 6 |
| D3YXF5 | C7 | 58.27 | 141.73 | 2.38E-03 | Complement component 7 |
| A0A0R4J032 | C9 | 65.80 | 134.20 | 1.47E-04 | Complement component 9 |
| P13634 | Ca1 | 75.00 | 125.00 | 4.12E-03 | Carbonic anhydrase 1 |
| E9PYN1 | Cadm1 | 61.63 | 138.40 | 7.14E-03 | Cell adhesion molecule 1 |
| Q8K354 | Cbr3 | 79.87 | 120.13 | 1.38E-03 | Carbonyl reductase [NADPH] 3 |
| A2APM2 | Cd44 | 78.87 | 121.13 | 3.27E-03 | CD44 antigen |
| Q9QWK4 | Cd5l | 43.63 | 156.37 | 2.71E-03 | CD5 antigen-like |
| A0A0R4J0G1 | Ceacam10 | 36.10 | 163.90 | 7.26E-02 | Carcinoembryonic antigen-related cell adhesion molecule 10 |
| Q9Z0H4 | Celf2 | 77.50 | 122.47 | 3.34E-03 | CUGBP Elav-like family member 2 |
| P23953 | Ces1c | 72.20 | 127.80 | 3.68E-05 | Carboxylesterase 1C |
| E9Q8I0 | Cfh | 67.73 | 132.23 | 2.48E-04 | Complement factor H |
| Q61406 | Cfhr1 | 58.50 | 141.50 | 1.19E-04 | Complement factor H-related 1 |
| Q4LDF6 | Cfhr2 | 63.77 | 136.23 | 2.15E-03 | Complement factor H-related 2 |
| P11680 | Cfp | 63.87 | 136.13 | 1.70E-04 | Properdin |
| O35744 | Chil3 | 67.67 | 132.37 | 7.06E-02 | Chitinase-like protein 3 |
| P60824 | Cirbp | 72.80 | 127.20 | 4.12E-02 | Cold-inducible RNA-binding protein |
| Q549A5 | Clu | 56.37 | 143.63 | 1.87E-04 | Clusterin |
| Q60847 | Col12a1 | 72.93 | 127.03 | 1.72E-06 | Collagen alpha-1(XII) chain |
| P28481 | Col2a1 | 76.80 | 123.17 | 1.98E-02 | Collagen alpha-1(II) chain |
| P02463 | Col4a1 | 77.77 | 122.23 | 7.08E-03 | Collagen alpha-1(IV) chain |
| Q9QZS0 | Col4a3 | 79.13 | 120.87 | 6.04E-03 | Collagen alpha-3(IV) chain |
| Q9QZR9 | Col4a4 | 61.73 | 138.30 | 2.33E-03 | Collagen alpha-4(IV) chain |
| Q63ZW6 | Col4a5 | 45.27 | 154.70 | 1.30E-04 | Col4a5 protein |
| B1AWB9 | Col5a1 | 63.03 | 136.97 | 6.41E-02 | Collagen, type V, alpha 1 |
| A0A087WS16 | Col6a3 | 79.83 | 120.13 | 4.38E-02 | Collagen, type VI, alpha 3 |
| E9Q6A6 | Col6a6 | 73.87 | 126.07 | 2.92E-03 | Collagen alpha-6(VI) chain |
| Q3T9L1 | Coro1a | 77.30 | 122.70 | 2.31E-04 | Coronin |
| P97315 | Csrp1 | 74.80 | 125.17 | 8.35E-02 | Cysteine and glycine-rich protein 1 |
| P97314 | Csrp2 | 78.37 | 121.63 | 1.19E-02 | Cysteine and glycine-rich protein 2 |
| Q9CX80 | Cygb | 66.07 | 133.93 | 1.54E-05 | Cytoglobin |
| P15392 | Cyp2a4 | 60.23 | 139.73 | 6.36E-07 | Cytochrome P450 2A4 |
| Q91X75 | Cyp2a5 | 44.77 | 155.27 | 1.87E-04 | Cyp2a4 protein |
| P11714 | Cyp2d9 | 78.23 | 121.73 | 6.02E-04 | Cytochrome P450 2D9 |
| Q91WL5 | Cyp4a12a | 69.60 | 130.40 | 1.75E-03 | Cytochrome P450 4A12A |
| A2A974 | Cyp4a12b | 70.70 | 129.30 | 1.53E-06 | Cytochrome P450, family 4, subfamily a, polypeptide 12B |
| Q3TSV1 | Dcn | 77.10 | 122.93 | 5.51E-02 | Decorin |
| Q80TN4 | Dnajc16 | 77.97 | 122.00 | 9.41E-03 | DnaJ homolog subfamily C member 16 |
| Q14BW6 | Dnase1 | 78.93 | 121.10 | 1.40E-02 | Deoxyribonuclease |
| Q8BPB5 | Efemp1 | 71.87 | 128.17 | 2.67E-03 | EGF-containing fibulin-like extracellular matrix protein 1 |
| Q3U3V1 | F10 | 78.17 | 121.83 | 2.51E-04 | Coagulation factor X |
| P16294 | F9 | 73.20 | 126.77 | 1.00E-03 | Coagulation factor IX |
| Q8K1B8 | Fermt3 | 69.97 | 130.00 | 1.10E-02 | Fermitin family homolog 3 |
| Q6YJU2 | Fetub | 67.70 | 132.30 | 5.45E-04 | GUGU alpha |
| Q543D7 | Fhl2 | 78.50 | 121.50 | 1.05E-02 | Four and a half LIM domains 2 |
| A0A087WSN6 | Fn1 | 79.73 | 120.23 | 2.14E-02 | Fibronectin |
| A2AKB4 | Frmpd1 | 74.97 | 125.00 | 8.27E-03 | FERM and PDZ domain-containing protein 1 |
| Q91XD4 | Ftcd | 72.77 | 127.23 | 1.38E-03 | Formimidoyltransferase-cyclodeaminase |
| P29391 | Ftl1 | 70.87 | 129.13 | 1.15E-02 | Ferritin light chain 1 |
| A2RSS9 | Ghrl | 71.17 | 128.87 | 7.07E-02 | Ghrelin |
| B1AVU4 | Gm14744 | 61.03 | 139.00 | 5.12E-02 | Predicted gene 14744 |
| E9Q035 | Gm20425 | 76.07 | 123.90 | 1.35E-03 | Predicted gene 20425 |
| E9PUM5 | Gm4788 | 79.33 | 120.67 | 4.03E-02 | Predicted gene 4788 |
| E9Q8B5 | Gm4788 | 74.57 | 125.43 | 1.43E-03 | Predicted gene 4788 |
| Q5I0T9 | Gnmt | 69.10 | 130.87 | 3.88E-03 | Glycine N-methyltransferase |
| Q8QZR5 | Gpt | 68.40 | 131.60 | 1.34E-05 | Alanine aminotransferase 1 |
| P46412 | Gpx3 | 77.43 | 122.57 | 9.49E-03 | Glutathione peroxidase 3 |
| P30115 | Gsta3 | 78.47 | 121.53 | 1.72E-04 | Glutathione S-transferase A3 |
| P48774 | Gstm5 | 79.47 | 120.53 | 2.01E-02 | Glutathione S-transferase Mu 5 |
| Q64471 | Gstt1 | 78.20 | 121.83 | 3.34E-03 | Glutathione S-transferase theta-1 |
| P01898 | H2-Q10 | 79.83 | 120.17 | 5.21E-03 | H-2 class I histocompatibility antigen, Q10 alpha chain |
| Q9NYQ2 | Hao2 | 75.80 | 124.17 | 1.06E-03 | Hydroxyacid oxidase 2 |
| A0A0E4B366 | HC | 29.57 | 170.43 | 8.43E-02 | MAb 44B1 heavy chain |
| P70349 | Hint1 | 79.17 | 120.87 | 9.40E-02 | Histidine triad nucleotide-binding protein 1 |
| P17095 | Hmga1 | 68.27 | 131.73 | 1.77E-02 | High mobility group protein HMG-I/HMG-Y |
| A2AQK4 | Hnmt | 76.27 | 123.70 | 2.05E-02 | Histamine N-methyltransferase |
| Q61646 | Hp | 56.03 | 144.00 | 1.22E-02 | Haptoglobin |
| P49429 | Hpd | 56.47 | 143.50 | 1.25E-03 | 4-hydroxyphenylpyruvate dioxygenase |
| Q91X72 | Hpx | 76.60 | 123.40 | 1.93E-03 | Hemopexin |
| Q9R118 | Htra1 | 50.17 | 149.83 | 5.93E-05 | Serine protease HTRA1 |
| Q9D236 | Htra3 | 51.33 | 148.67 | 3.30E-03 | Serine protease HTRA3 |
| Q4FJY6 | Ifit3 | 78.87 | 121.13 | 2.03E-03 | Ifit3 protein |
| O88477 | Igf2bp1 | 79.23 | 120.73 | 2.97E-02 | Insulin-like growth factor 2 mRNA-binding protein 1 |
| Q5F2I8 | IgG1 | 35.17 | 164.83 | 4.73E-03 | Gamma heavy chain variable region (Fragment) |
| Q6PIP8 | Igh | 58.23 | 141.77 | 3.22E-02 | Igh protein |
| Q80ZI7 | Igh | 37.53 | 162.47 | 3.66E-03 | Igh protein |
| Q8K0Z4 | Igh | 45.17 | 154.80 | 1.92E-05 | Igh protein |
| Q91Z05 | Ighg | 38.93 | 161.03 | 7.82E-03 | Ighg protein |
| A0A1Y7VJN6 | Ighg3 | 54.43 | 145.57 | 5.91E-02 | Immunoglobulin heavy constant gamma 3 |
| A0A075B5P6 | Ighm | 29.33 | 170.63 | 2.77E-02 | Immunoglobulin heavy constant mu |
| A0A0A6YXN4 | Ighv1-18 | 39.77 | 160.23 | 3.83E-03 | Immunoglobulin heavy variable V1-18 |
| A0A075B5V0 | Ighv1-26 | 44.53 | 155.50 | 1.40E-02 | Immunoglobulin heavy variable 1-26 |
| A0A075B5V1 | Ighv1-31 | 44.13 | 155.87 | 1.20E-03 | Immunoglobulin heavy variable 1-31 |
| A0A075B5W5 | Ighv1-54 | 44.50 | 155.50 | 1.18E-02 | Immunoglobulin heavy variable V1-54 |
| X5J4V1 | Ighv1-62 | 51.03 | 148.93 | 5.71E-02 | IgA heavy chain VDJ region |
| A0A0G2JFE9 | Ighv1-76 | 66.80 | 133.20 | 4.89E-02 | Immunoglobulin heavy variable 1-76 |
| A0A075B5Y4 | Ighv1-81 | 26.60 | 173.40 | 1.07E-02 | Immunoglobulin heavy variable 1-81 |
| P18531 | Ighv3-6 | 33.17 | 166.83 | 9.10E-02 | Ig heavy chain V region 3-6 |
| A0A075B5R5 | Ighv4-1 | 56.70 | 143.30 | 5.10E-02 | Immunoglobulin heavy variable 4-1 |
| A0A0B4J1P4 | Ighv5-16 | 65.03 | 135.00 | 5.62E-04 | Immunoglobulin heavy variable 5-16 |
| J3QK03 | Ighv6-5 | 32.13 | 167.87 | 1.34E-02 | Immunoglobulin heavy variable V6-5 |
| A0A0A6YXQ0 | Ighv8-8 | 42.33 | 157.67 | 5.51E-02 | Immunoglobulin heavy variable 8-8 |
| I6L958 | Igk | 73.93 | 126.07 | 2.23E-03 | Igk protein |
| A2NHM3 | Igkc | 31.07 | 168.97 | 9.40E-04 | If kappa light chain |
| A0A140T8M2 | Igkv12-44 | 48.37 | 151.67 | 9.26E-03 | Immunoglobulin kappa variable 12-44 |
| P01633 | Igkv19-17 | 57.50 | 142.53 | 3.27E-02 | Ig kappa chain V19-17 |
| A0A0B4J1I9 | Igkv4-55 | 39.40 | 160.63 | 9.06E-02 | Immunoglobulin kappa variable 4-55 |
| A0A075B5M1 | Igkv4-63 | 66.67 | 133.33 | 8.87E-02 | Immunoglobulin kappa variable 4-63 |
| A0A140T8M5 | Igkv6-15 | 61.83 | 138.17 | 2.40E-02 | Immunoglobulin kappa variable 6-15 |
| A0A0G2JEI6 | Igkv6-29 | 34.47 | 165.53 | 2.54E-02 | Immunoglobulin kappa chain variable 6-29 |
| A0A075B5K2 | Igkv9-124 | 43.17 | 156.83 | 1.32E-02 | Immunoglobulin kappa chain variable 9-124 |
| Q7TMY7 | Ipo8 | 79.63 | 120.37 | 2.98E-03 | Importin-8 |
| A6X935 | Itih4 | 76.53 | 123.47 | 3.13E-03 | Inter alpha-trypsin inhibitor, heavy chain 4 |
| P01592 | Jchain | 54.33 | 145.67 | 5.37E-05 | Immunoglobulin J chain |
| A0A0R4J038 | Kng1 | 69.57 | 130.47 | 5.89E-03 | Kininogen-1 |
| Q9D312 | Krt20 | 76.07 | 123.93 | 1.40E-02 | Keratin, type I cytoskeletal 20 |
| Q8BTY1 | Kyat1 | 78.83 | 121.17 | 9.12E-04 | Kynurenine--oxoglutarate transaminase 1 |
| A2ARD6 | Kynu | 74.23 | 125.73 | 1.36E-03 | Kynureninase |
| Q9Z175 | Loxl3 | 71.00 | 129.03 | 4.41E-03 | Lysyl oxidase homolog 3 |
| Q91XL1 | Lrg1 | 72.57 | 127.43 | 2.44E-02 | Leucine-rich HEV glycoprotein |
| P19973 | Lsp1 | 79.60 | 120.43 | 4.35E-03 | Lymphocyte-specific protein 1 |
| O08999 | Ltbp2 | 65.87 | 134.10 | 1.03E-02 | Latent-transforming growth factor beta-binding protein 2 |
| P51885 | Lum | 74.50 | 125.53 | 1.22E-02 | Lumican |
| O88188 | Ly86 | 69.63 | 130.40 | 1.15E-02 | Lymphocyte antigen 86 |
| P08905 | Lyz2 | 75.50 | 124.50 | 1.88E-03 | Lysozyme C-2 |
| P98064 | Masp1 | 66.13 | 133.87 | 2.98E-02 | Mannan-binding lectin serine protease 1 |
| Q91WP0 | Masp2 | 78.47 | 121.57 | 7.12E-03 | Mannan-binding lectin serine protease 2 |
| P39039 | Mbl1 | 77.17 | 122.83 | 3.72E-04 | Mannose-binding protein A |
| B0R0U1 | Mettl8 | 79.07 | 120.93 | 1.31E-02 | Methyltransferase-like protein |
| P21956 | Mfge8 | 52.43 | 147.57 | 3.70E-02 | Lactadherin |
| P19788 | Mgp | 68.57 | 131.40 | 4.03E-02 | Matrix Gla protein |
| Q62000 | Mim | 70.87 | 129.13 | 8.95E-05 | Mimecan |
| P0DOV1 | Mnda | 77.67 | 122.33 | 2.80E-05 | Interferon-activable protein 205-B |
| P02802 | Mt1 | 67.30 | 132.67 | 2.87E-02 | Metallothionein-1 |
| P02798 | Mt2 | 58.53 | 141.50 | 1.80E-02 | Metallothionein-2 |
| Q9D8I1 | Mzb1 | 25.20 | 174.83 | 5.01E-02 | Marginal zone B- and B1-cell-specific protein |
| Q8R007 | Nectin4 | 77.13 | 122.87 | 1.84E-02 | Nectin-4 |
| P19246 | Nefh | 77.53 | 122.50 | 1.23E-02 | Neurofilament heavy polypeptide |
| Q61982 | Notch3 | 77.50 | 122.50 | 1.39E-02 | Neurogenic locus notch homolog protein 3 |
| Q0VEM1 | Ntn4 | 66.57 | 133.47 | 5.09E-05 | Netrin 4 |
| Q61205 | Pafah1b3 | 73.97 | 126.03 | 3.96E-04 | Platelet-activating factor acetylhydrolase IB subunit gamma |
| B7ZN28 | Papln | 79.20 | 120.80 | 3.29E-04 | Papilin |
| Q3ULW8 | Parp3 | 70.97 | 129.00 | 2.29E-04 | Poly [ADP-ribose] polymerase |
| Q09098 | Pate4 | 28.90 | 171.10 | 9.17E-03 | Prostate and testis expressed protein 4 |
| O88502 | Pde8a | 64.93 | 135.03 | 3.92E-02 | High affinity cAMP-specific and IBMX-insensitive 3',5'-cyclic phosphodiesterase 8A |
| Q6P8R3 | Pf4 | 63.87 | 136.13 | 1.11E-02 | C-X-C motif chemokine |
| Q80TL4 | Phf24 | 71.40 | 128.60 | 2.31E-04 | PHD finger protein 24 |
| O70570 | Pigr | 55.07 | 144.93 | 3.74E-06 | Polymeric immunoglobulin receptor |
| P55065 | Pltp | 64.13 | 135.83 | 2.22E-02 | Phospholipid transfer protein |
| Q7TQ62 | Podn | 72.90 | 127.10 | 4.32E-03 | Podocan |
| Q9EQI5 | Ppbp | 57.33 | 142.63 | 5.82E-04 | Chemokine (C-X-C motif) ligand 7, isoform CRA_b |
| G3X981 | Prph | 76.00 | 124.00 | 1.92E-05 | Peripherin |
| O35955 | Psmb10 | 77.83 | 122.10 | 1.28E-02 | Proteasome subunit beta type-10 |
| Q8K426 | Retnlg | 73.90 | 126.10 | 5.91E-04 | Resistin-like gamma |
| Q7TT28 | Rexo1 | 78.00 | 121.97 | 1.66E-02 | RNA exonuclease 1 homolog |
| Q32MD7 | Rgs10 | 64.13 | 135.87 | 5.04E-04 | Regulator of G-protein signalling 10 |
| Q9JJC6 | Rilpl1 | 79.90 | 120.13 | 2.64E-02 | RILP-like protein 1 |
| P59729 | Rin3 | 77.20 | 122.83 | 1.11E-02 | Ras and Rab interactor 3 |
| E9Q7F2 | Rnf169 | 77.50 | 122.50 | 2.67E-02 | E3 ubiquitin-protein ligase RNF169 |
| Q9CQR2 | Rps21 | 77.23 | 122.77 | 3.41E-02 | 40S ribosomal protein S21 |
| P62274 | Rps29 | 54.43 | 145.53 | 3.09E-02 | 40S ribosomal protein S29 |
| P07091 | S100a4 | 70.07 | 129.93 | 1.98E-03 | Protein S100-A4 |
| A0A3B2WCG9 | Scube3 | 67.57 | 132.40 | 4.67E-04 | Signal peptide, CUB and EGF-like domain-containing protein 3 |
| O08992 | Sdcbp | 70.63 | 129.37 | 2.43E-03 | Syntenin-1 |
| A0A0R4J135 | Selenbp2 | 76.67 | 123.30 | 1.05E-02 | Selenium-binding protein 2 |
| Q8R121 | Serpina10 | 59.67 | 140.30 | 7.07E-03 | Protein Z-dependent protease inhibitor |
| P49182 | Serpind1 | 79.83 | 120.13 | 1.38E-03 | Heparin cofactor 2 |
| Q07235 | Serpine2 | 76.37 | 123.67 | 2.78E-02 | Glia-derived nexin |
| E9Q5F9 | Setd2 | 77.33 | 122.67 | 2.29E-02 | Histone-lysine N-methyltransferase SETD2 |
| Q91VW3 | Sh3bgrl3 | 73.60 | 126.40 | 3.02E-02 | SH3 domain-binding glutamic acid-rich-like protein 3 |
| A0A0R4J1V1 | Slc39a14 | 73.57 | 126.43 | 3.76E-02 | Solute carrier family 39 (Zinc transporter), member 14, isoform CRA_a |
| Q5GQ64 | Sncg | 76.83 | 123.17 | 4.51E-02 | Gamma-synuclein (Fragment) |
| Q8K1I3 | Spp2 | 67.13 | 132.87 | 5.54E-02 | Secreted phosphoprotein 24 |
| Q3UN54 | Sslp1 | 48.33 | 151.70 | 2.31E-04 | Secreted seminal-vesicle Ly-6 protein 1 |
| B7ZNH4 | Stk-ps2 | 65.57 | 134.40 | 2.78E-02 | EG212225 protein |
| Q9D939 | Sult1c2 | 74.33 | 125.70 | 5.46E-05 | Sulfotransferase 1C2 |
| E9PX96 | Sva | 44.43 | 155.57 | 2.17E-02 | Seminal vesicle antigen |
| Q6WIZ7 | Svs1 | 52.33 | 147.67 | 4.10E-02 | Amine oxidase |
| F2Z472 | Svs3a | 45.87 | 154.13 | 1.91E-02 | Seminal vesicle secretory protein 3A |
| P18419 | Svs4 | 32.23 | 167.77 | 7.81E-02 | Seminal vesicle secretory protein 4 |
| P30933 | Svs5 | 40.10 | 159.93 | 1.39E-02 | Seminal vesicle secretory protein 5 |
| P37804 | Tagln | 77.40 | 122.60 | 1.05E-03 | Transgelin |
| Q9WVA4 | Tagln2 | 79.40 | 120.57 | 7.23E-03 | Transgelin-2 |
| Q921I1 | Tf | 75.37 | 124.63 | 6.05E-03 | Serotransferrin |
| A1L353 | Tgfbi | 65.33 | 134.63 | 9.87E-05 | Transforming growth factor, beta induced |
| Q923W1 | Tgs1 | 79.23 | 120.77 | 9.13E-02 | Trimethylguanosine synthase |
| Q80YQ1 | Thbs1 | 64.07 | 135.93 | 3.50E-05 | Thrombospondin-1 |
| Q54AE5 | Timp3 | 75.57 | 124.43 | 9.41E-05 | Brain cDNA, clone MNCb-5810, similar to Mus musculus tissue inhibitor of metalloproteinase 3 (Timp3), mRNA |
| P20065 | Tmsb4x | 75.03 | 124.97 | 5.37E-02 | Thymosin beta-4 |
| Q80YX1 | Tnc | 72.63 | 127.37 | 9.43E-03 | Tenascin |
| O35452 | Tnxb | 78.70 | 121.27 | 1.43E-04 | Tenascin X |
| A0A0R4J018 | Tpmt | 74.00 | 126.03 | 9.77E-03 | Thiopurine S-methyltransferase |
| B7ZNI7 | Treml1 | 68.73 | 131.30 | 1.22E-03 | Treml1 protein |
| E9PY03 | Tstd1 | 62.50 | 137.53 | 4.00E-02 | Thiosulfate sulfurtransferase (rhodanese)-like domain-containing 1 |
| P07309 | Ttr | 77.63 | 122.40 | 2.27E-02 | Transthyretin |
| Q9QZM0 | Ubqln2 | 67.60 | 132.40 | 3.58E-02 | Ubiquilin-2 |
| Q9R0P9 | Uchl1 | 75.37 | 124.60 | 1.12E-03 | Ubiquitin carboxyl-terminal hydrolase isozyme L1 |
| Q91X17 | Umod | 53.47 | 146.53 | 5.20E-04 | Uromodulin |
| P29788 | Vtn | 68.83 | 131.17 | 1.08E-04 | Vitronectin |
| Q8K1I7 | Wipf1 | 74.00 | 126.03 | 9.48E-03 | WAS/WASL-interacting protein family member 1 |
| E9Q444 | Zbtb21 | 79.93 | 120.07 | 2.36E-02 | Zinc finger and BTB domain-containing 21 |
| Q91X58 | Zfand2b | 75.77 | 124.20 | 3.53E-02 | AN1-type zinc finger protein 2B |
