# Supplementary material for: Comparative proteomic analysis identifies biomarkers for renal aging
Source: Aging (Albany NY). 2020 Nov 6;12(21):21890–903. doi: 10.18632/aging.104007 (PMC7695359; doi:10.18632/aging.104007)
Supplement: Supplementary Table 2 [file aging-12-104007-s002..docx]

**Supplementary Table 2. The downregulated proteins in aged kidney.**

| Accession | Gene Symbol | Kidney Young | Kidney Aged | p-value | Description |
| --- | --- | --- | --- | --- | --- |
| P55096 | Abcd3 | 118.63 | 81.33 | 2.97E-02 | ATP-binding cassette sub-family D member 3 |
| P45952 | Acadm | 118.63 | 81.37 | 8.94E-04 | Medium-chain specific acyl-CoA dehydrogenase, mitochondrial |
| A2AKK5 | Acnat1 | 121.43 | 78.57 | 2.97E-02 | Acyl-coenzyme A amino acid acyltransferase 1 |
| Q8BWN8 | Acot4 | 118.53 | 81.43 | 8.03E-03 | Acyl-coenzyme A thioesterase 4 |
| Q3U965 | Acot8 | 120.17 | 79.83 | 7.62E-04 | Acyl-CoA thioesterase 8 |
| Q9R0H0 | Acox1 | 123.23 | 76.77 | 9.13E-04 | Peroxisomal acyl-coenzyme A oxidase 1 |
| Q9QXD1 | Acox2 | 130.23 | 69.77 | 1.90E-02 | Peroxisomal acyl-coenzyme A oxidase 2 |
| Q91VA0 | Acsm1 | 118.97 | 81.03 | 1.09E-02 | Acyl-coenzyme A synthetase ACSM1 |
| D3Z0L8 | Actn4 | 158.53 | 41.47 | 3.13E-02 | Alpha-actinin-4 |
| A2AL50 | Agps | 117.43 | 82.57 | 1.49E-02 | Alkylglycerone-phosphate synthase |
| A0A1L1SQE2 | Ahi1 | 128.83 | 71.20 | 3.17E-02 | Jouberin |
| F7BP55 | Ak2 | 133.60 | 66.40 | 2.82E-04 | Adenylate kinase 2, mitochondrial |
| Q8K023 | Akr1c18 | 149.70 | 50.33 | 8.59E-04 | Aldo-keto reductase family 1 member C18 |
| O09174 | Amacr | 118.00 | 81.97 | 4.30E-04 | Alpha-methylacyl-CoA racemase |
| D3YX85 | Asap2 | 129.50 | 70.53 | 4.70E-02 | Arf-GAP with SH3 domain, ANK repeat and PH domain-containing protein 2 |
| Q6ZQ17 | Atp11b | 122.93 | 77.07 | 2.20E-02 | Phospholipid-transporting ATPase |
| G3UW46 | Atp23 | 118.13 | 81.87 | 1.46E-04 | Mitochondrial inner membrane protease ATP23 |
| Q99L60 | Atp6v1c2 | 119.57 | 80.47 | 5.62E-02 | V-type proton ATPase subunit C 2 |
| Q8BMC1 | Atp6v1g3 | 121.33 | 78.63 | 1.23E-02 | V-type proton ATPase subunit G 3 |
| O08734 | Bak1 | 118.43 | 81.57 | 3.89E-02 | Bcl-2 homologous antagonist/killer |
| Q6QD59 | Bnip1 | 119.03 | 80.97 | 4.31E-02 | Vesicle transport protein SEC20 |
| P12658 | Calb1 | 124.07 | 75.93 | 1.94E-05 | Calbindin |
| Q9DC50 | Carn | 138.47 | 61.57 | 5.89E-04 | Peroxisomal carnitine |
| P24270 | Cat | 119.27 | 80.80 | 1.21E-02 | Catalase |
| P60334 | Cdo1 | 121.47 | 78.53 | 1.69E-03 | Cysteine dioxygenase type 1 |
| Q5FWI3 | Cemip2 | 127.13 | 72.87 | 4.49E-02 | Cell surface hyaluronidase |
| Q6PDB7 | Ces2b | 121.67 | 78.37 | 1.20E-02 | Carboxylic ester hydrolase |
| D3YU39 | Chpt1 | 139.00 | 61.00 | 3.33E-02 | Cholinephosphotransferase 1 |
| Q8VDH2 | Cinp | 155.53 | 44.47 | 3.96E-02 | 2810452K22Rik protein |
| Q99LI9 | Clp1 | 132.13 | 67.87 | 3.28E-02 | Polyribonucleotide 5'-hydroxyl-kinase Clp1 |
| O35206 | Col15a1 | 120.80 | 79.20 | 2.29E-03 | Collagen alpha-1(XV) chain |
| B1AVH5 | Coro2a | 119.47 | 80.50 | 3.13E-02 | Coronin |
| Q8K0C4 | Cyp51a1 | 117.37 | 82.63 | 2.18E-02 | Lanosterol 14-alpha demethylase |
| Q3UQE2 | Defb1 | 123.70 | 76.30 | 3.05E-02 | Defensin beta 1 |
| Q8R0K9 | E2f4 | 117.83 | 82.10 | 3.77E-02 | Transcription factor E2F4 |
| Q78JN3 | Eci3 | 124.67 | 75.37 | 8.71E-08 | Enoyl-CoA delta isomerase 3, peroxisomal |
| Q9DBM2 | Ehhadh | 130.73 | 69.27 | 1.97E-04 | Peroxisomal bifunctional enzyme |
| Q7TSV9 | Enkd1 | 134.63 | 65.37 | 6.59E-03 | Enkurin domain-containing protein 1 |
| D0VYV6 | Epb41l3 | 123.67 | 76.30 | 1.54E-02 | Band 4.1-like protein 3 |
| Q9QXB3 | FBP1 | 132.83 | 67.10 | 2.72E-02 | Fructose-1,6-bisphosphatase (Fragment) |
| Q8BG80 | Fbxo46 | 120.23 | 79.73 | 2.11E-02 | F-box only protein 46 |
| A2AFQ9 | Gemin5 | 119.33 | 80.63 | 4.10E-02 | Gem-associated protein 5 |
| A0A1B0GRV7 | Gm17949 | 128.83 | 71.13 | 3.91E-02 | Predicted gene, 17949 |
| Q5U4C1 | Gprasp1 | 187.80 | 12.23 | 4.67E-02 | G-protein coupled receptor-associated sorting protein 1 |
| Q9QXE0 | Hacl1 | 117.83 | 82.13 | 4.17E-02 | 2-hydroxyacyl-CoA lyase 1 |
| P43276 | Hist1h1b | 141.93 | 58.07 | 8.02E-03 | Histone H1.5 |
| P43274 | Hist1h1e | 117.80 | 82.20 | 3.19E-02 | Histone H1.4 |
| Q8CGP5 | Hist1h2af | 141.37 | 58.63 | 4.79E-02 | Histone H2A type 1-F |
| Q6NSP9 | Hmga2 | 154.87 | 45.10 | 1.36E-04 | High mobility group protein HMGI-C |
| P51660 | Hsd17b4 | 119.73 | 80.30 | 3.83E-04 | Peroxisomal multifunctional enzyme type 2 |
| Q9EQD6 | K16 | 118.80 | 81.20 | 2.81E-02 | Keratin intermediate filament 16a |
| O35738 | Klf12 | 119.23 | 80.77 | 5.96E-02 | Krueppel-like factor 12 |
| P15947 | Klk1 | 133.50 | 66.50 | 2.81E-03 | Kallikrein-1 |
| B2RTP7 | Krt2 | 124.77 | 75.23 | 2.27E-02 | Krt2 protein |
| Q32P04 | Krt5 | 120.97 | 79.07 | 5.57E-02 | Keratin 5 |
| Q6NXH9 | Krt73 | 132.50 | 67.53 | 2.17E-02 | Keratin, type II cytoskeletal 73 |
| Q3UV17 | Krt76 | 124.37 | 75.63 | 3.50E-02 | Keratin, type II cytoskeletal 2 oral |
| E9Q0F0 | Krt78 | 134.00 | 66.03 | 3.33E-02 | Keratin 78 |
| Q8VED5 | Krt79 | 125.87 | 74.13 | 2.95E-02 | Keratin, type II cytoskeletal 79 |
| Q61789 | Lama3 | 119.47 | 80.57 | 4.22E-02 | Laminin subunit alpha-3 |
| Q3U2W5 | Lgals8 | 122.33 | 77.67 | 3.48E-02 | Galectin |
| Q9DBN5 | Lonp2 | 123.23 | 76.73 | 7.83E-04 | Lon protease homolog 2, peroxisomal |
| G5E8Y2 | Lrrcc1 | 155.87 | 44.17 | 3.42E-02 | Leucine rich repeat and coiled-coil domain containing 1 |
| A2CEL1 | Mup1 | 124.73 | 75.30 | 3.48E-02 | Major urinary protein 1 |
| A2AKN8 | Mup8 | 117.90 | 82.10 | 3.68E-02 | Major urinary protein 5 |
| F8VQL9 | Ncor2 | 118.23 | 81.77 | 2.39E-02 | Nuclear receptor corepressor 2 |
| A0A2R8VHX1 | Ndufa6 | 122.03 | 78.00 | 4.42E-02 | NADH dehydrogenase [ubiquinone] 1 alpha subcomplex subunit 6 |
| E9Q8I7 | Nfxl1 | 153.43 | 46.53 | 3.81E-02 | Nuclear transcription factor, X-box-binding-like 1 |
| P11930 | Nudt19 | 119.97 | 80.03 | 7.73E-04 | Nucleoside diphosphate-linked moiety X motif 19 |
| A0A0R4J0G5 | Parpbp | 125.37 | 74.63 | 2.55E-02 | PCNA-interacting partner |
| Q8CI37 | Pck1 | 121.83 | 78.20 | 4.91E-02 | Phosphoenolpyruvate carboxykinase 1, cytosolic |
| Q99MZ7 | Pecr | 118.63 | 81.37 | 2.26E-03 | Peroxisomal trans-2-enoyl-CoA reductase |
| P0DJE0 | Pet100 | 117.83 | 82.17 | 3.08E-02 | Protein PET100 homolog, mitochondrial |
| Q8R2H9 | Phospho1 | 141.93 | 58.10 | 4.71E-02 | Phosphoethanolamine/phosphocholine phosphatase |
| B2RPU2 | Plekhd1 | 122.23 | 77.73 | 8.44E-04 | Pleckstrin homology domain-containing family D member 1 |
| Q62087 | Pon3 | 119.27 | 80.77 | 1.09E-02 | Serum paraoxonase/lactonase 3 |
| Q8R3K3 | Ptcd2 | 118.93 | 81.07 | 2.02E-02 | Pentatricopeptide repeat-containing protein 2, mitochondrial |
| P35831 | Ptpn12 | 167.87 | 32.17 | 4.00E-02 | Tyrosine-protein phosphatase non-receptor type 12 |
| A0A2I3BPN8 | Ralgapa2 | 118.03 | 81.97 | 3.20E-02 | Ral GTPase-activating protein subunit alpha-2 |
| A0A0A6YG7 | Rapgef2 | 120.90 | 79.10 | 4.28E-02 | Rap guanine nucleotide exchange factor 2 |
| P35230 | Reg3b | 130.37 | 69.63 | 3.19E-04 | Regenerating islet-derived protein 3-beta |
| Q545I1 | Reg3g | 119.40 | 80.57 | 1.45E-03 | Regenerating islet-derived 3 gamma |
| P06281 | Ren1 | 138.33 | 61.70 | 2.09E-02 | Renin-1 |
| G3UWX1 | Rfc1 | 123.47 | 76.53 | 4.22E-02 | Replication factor C subunit 1 |
| Q5I0T8 | Rpl19 | 122.67 | 77.30 | 3.31E-02 | Ribosomal protein L19 |
| Q6ZWV7 | Rpl35 | 119.17 | 80.83 | 3.57E-02 | 60S ribosomal protein L35 |
| P83882 | Rpl36a | 119.80 | 80.23 | 3.34E-02 | 60S ribosomal protein L36a |
| Q8K1J5 | Sde2 | 125.43 | 74.57 | 4.70E-02 | Replication stress response regulator SDE2 |
| Q3U9N9 | Slc16a10 | 131.13 | 68.87 | 5.20E-02 | Monocarboxylate transporter 10 |
| Q8VCA0 | Slc22a19 | 136.63 | 63.37 | 3.12E-02 | Solute carrier family 22 member 19 |
| A0A0R4J122 | Slc22a7 | 125.80 | 74.17 | 1.78E-02 | Solute carrier family 22 member 7 |
| B0LAB5 | Slc25a5 | 152.77 | 47.23 | 4.05E-02 | Solute carrier family 25 member 5 |
| P14246 | Slc2a2 | 120.10 | 79.87 | 4.09E-02 | Solute carrier family 2, facilitated glucose transporter member 2 |
| P10852 | Slc3a2 | 122.93 | 77.07 | 2.56E-02 | 4F2 cell-surface antigen heavy chain |
| O88343 | Slc4a4 | 117.37 | 82.60 | 9.10E-03 | Electrogenic sodium bicarbonate cotransporter 1 |
| E9Q3M5 | Slc4a5 | 122.53 | 77.43 | 7.53E-03 | Anion exchange protein |
| Q9QXW9 | Slc7a8 | 122.57 | 77.43 | 3.27E-02 | Large neutral amino acids transporter small subunit 2 |
| G3X9J1 | Slc8a1 | 121.93 | 78.10 | 4.92E-02 | Sodium/calcium exchanger 1 |
| Q3V460 | Smim26 | 119.90 | 80.07 | 5.17E-02 | Gene model 561 |
| Q69ZV4 | Ssx2ip | 122.67 | 77.37 | 5.98E-02 | MKIAA0923 protein |
| Q9D0I4 | Stx17 | 124.63 | 75.40 | 4.37E-02 | Syntaxin-17 |
| E9PUP1 | Syde2 | 149.80 | 50.17 | 2.43E-02 | Synapse defective 1, Rho GTPase |
| F6SEU4 | Syngap1 | 130.03 | 69.93 | 4.03E-02 | Ras/Rap GTPase-activating protein SynGAP |
| A0A0R4J293 | Tgm1 | 118.30 | 81.70 | 1.55E-05 | Protein-glutamine gamma-glutamyltransferase K |
| Q7TSH8 | Tmem94 | 129.27 | 70.73 | 3.77E-02 | Transmembrane protein 94 |
| A2ASS6 | Ttn | 125.67 | 74.33 | 4.08E-03 | Titin |
| D5MR34 | Tubb3 | 126.63 | 73.37 | 3.10E-02 | Tubulin beta chain |
| Q3TTF2 | Ubxn8 | 124.70 | 75.30 | 4.65E-02 | UBX domain-containing protein 8 |
| B1AVD2 | Xpnpep2 | 123.77 | 76.27 | 7.83E-03 | Xaa-Pro aminopeptidase 2 |
| Q9Z2C8 | Ybx2 | 118.57 | 81.43 | 5.39E-04 | Y-box-binding protein 2 |
| Q0VGT4 | Zgrf1 | 118.57 | 81.43 | 1.57E-02 | Protein ZGRF1 |
