# Supplementary material for: Comparative proteomic analysis identifies biomarkers for renal aging
Source: Aging (Albany NY). 2020 Nov 6;12(21):21890–903. doi: 10.18632/aging.104007 (PMC7695359; doi:10.18632/aging.104007)
Supplement: Supplementary Tables 3 and 4 [file aging-12-104007-s003..pdf]

SUPPLEMENTARY TABLES

Supplementary Table 3. The location of biomarkers for renal aging.

Accession	Gene Name	Description	Ratio (Aged/Young)	Location
Q9Z2V4	Pck1	Phosphoenolpyruvate carboxykinase	0.58	Proximal tubule brush border cells; Proximal tubule epithelial cells
P12658	Calb1	Calbindin	0.71	Distal convoluted tubule cells; Principal cells; Renal connecting tubule
P32020	Scp2	Non-specific lipid-transfer protein	0.72	Proximal tubule brush border cells;
P97494	Gclc	Glutamate--cysteine ligase catalytic subunit	0.72	S3 proximal tubule cells;
P24270	Cat	Catalase	0.70	Proximal tubule brush border cells; Proximal tubule cells;
P06281	Ren1	Renin-1	0.58	Metanephros; Juxtaglomerular cells
Q64433	Hspe1	10 kDa heat shock protein	0.73	Fenestrated endothelial cells; Proximal tubule cells;
Q9JKR6	Hyou1	Hypoxia up-regulated protein 1	0.76	
A2A7A7	H6pd	GDH/6PGL endoplasmic bifunctional protein	1.36	The p3 segment of proximal convolutions and collecting tubles in the cortex and inner medulla of the kidney
Q91X72	Hpx	Hemopexin	1.44	Plasma protein;
P13597	Icam1	Intercellular adhesion molecule 1	1.26	Fenestrated endothelial cells; Principal cells; Stromal cells;
P09813	Apoa2	Apolipoprotein A-II	1.75	Plasma protein;
P30115	Gsta3	Glutathione S-transferase A3	1.51	Proximal tubule brush border cells;
Q00623	Apoa1	Apolipoprotein A-I	1.34	Major protein of plasma HDL;
P46412	Gpx3	Glutathione peroxidase 3	1.91	Plasma protein;
P18242	Ctsd	Cathepsin D	1.49	Epithelial cells; Intercalated cells; Principal cells; Stromal cells; Urothelial cells;
Q07797	Lgals3bp	Galectin-3-binding protein	1.66	Stromal cells;
Q549A5	Clu	Clusterin	2.00	Distal convoluted tubule cells; Epithelial cells; Principal cells; Stromal cells; Urothelial cells
P21614	Gc	Vitamin D-binding protein	2.25	Stromal cells;
P08226	Apoe	Apolipoprotein E	1.93	Proximal tubule cells; S1 proximal tubule cells; Stromal cells
A2ATU0	Dhtkd1	Probable 2-oxoglutarate dehydrogenase E1 component DHKTD1	1.90	
O70570	Pigr	Polymeric immunoglobulin receptor	2.23	The epithelial cells of the distal urinary tubule and of Henle’s loop
P29788	Vtn	Vitronectin	2.02	Plasma protein
Q91X17	Umod	Uromodulin	2.14	Ascending loop of Henle; Distal convoluted tubule cells; Epithelial cells; distal straight tubule and thin ascending limb of loop of Henle
P24549	Aldh1a1	Retinal dehydrogenase 1	2.56	In the medullary thick ascending limb segment of the loop of Henle
Q8K1I3	Spp2	Secreted phosphoprotein 24	2.52	Proximal tubule cells; S1 proximal tubule cells;
Q9QWK4	Cd5l	CD5 antigen-like	3.33	

Supplementary Table 4. NMN reversement of biomarkers for renal aging.

Accession	Gene Name	Description	Ratio(Aged/Young)	Ratio(Aged+NMN/Aged)
Q9Z2V4	Pck1	Phosphoenolpyruvate carboxykinase, cytosolic [GTP]	0.58	1.28
P12658	Calb1	Calbindin	0.71	1.29
P32020	Scp2	Non-specific lipid-transfer protein	0.72	0.99
P97494	Gclc	Glutamate--cysteine ligase catalytic subunit	0.72	1.22
P24270	Cat	Catalase	0.70	1.34
P06281	Ren1	Renin-1	0.58	1.30
Q64433	Hspe1	10 kDa heat shock protein	0.73	1.28
Q9JKR6	Hyou1	Hypoxia up-regulated protein 1	0.76	0.97
A2A7A7	H6pd	GDH/6PGL endoplasmic bifunctional protein	1.36	0.77
Q91X72	Hpx	Hemopexin	1.44	0.78
P13597	Icam1	Intercellular adhesion molecule 1	1.26	0.88
P09813	Apoa2	Apolipoprotein A-II	1.75	0.83
P30115	Gsta3	Glutathione S-transferase A3	1.51	0.95
Q00623	Apoa1	Apolipoprotein A-I	1.34	1.09
P46412	Gpx3	Glutathione peroxidase 3	1.91	0.76
P18242	Ctsd	Cathepsin D	1.49	0.62
Q07797	Lgals3bp	Galectin-3-binding protein	1.66	0.85
Q549A5	Clu	Clusterin	2.00	0.62
P21614	Gc	Vitamin D-binding protein	2.25	0.85
P08226	Apoe	Apolipoprotein E	1.93	1.11
A2ATU0	Dhtkd1	Probable 2-oxoglutarate dehydrogenase E1 component DHKTD1	1.90	1.10
O70570	Pigr	Polymeric immunoglobulin receptor	2.23	0.83
P29788	Vtn	Vitronectin	2.02	0.71
Q91X17	Umod	Uromodulin	2.14	0.42
P24549	Aldh1a1	Retinal dehydrogenase 1	2.56	0.57
Q8K1I3	Spp2	Secreted phosphoprotein 24	2.52	0.58
Q9QWK4	Cd5l	CD5 antigen-like	3.33	0.68
